# Temperature dependence for high electrical performance of Mn-doped high surface area activated carbon (HSAC) as additives for hybrid capacitor

**DOI:** 10.1038/s41598-020-79469-7

**Published:** 2021-01-12

**Authors:** Zambaga Otgonbayar, Kamrun Nahar Fatema, Sunhye Yang, Ick-Jun Kim, Minchul Kim, Sang Eun Shim, Won-Chun Oh

**Affiliations:** 1 Department of Advanced Materials Science and Engineering, Hanseo University, Seosan-si, Chungnam 356-706 Republic of Korea; 2 College of Materials Science and Engineering, Anhui University of Science and Technology, Huainan, 232001 People’s Republic of China; 3 Korea Electrotechnology Research Institute, 12, Boolmosan-ro, 10beon-gil, Seongsan-gu, Changwon-si, Gyeongsangnam-do 51543 Republic of Korea; 4 Department of Chemical Engineering, Inha University, 100 Inha-ro, Michuhol-gu, Incheon, 22212 Republic of Korea

**Keywords:** Energy science and technology, Materials science

## Abstract

Herein, we manufactured the positive and negative electrodes for the hybrid capacitor. The Mn-doped High surface area of Activated carbon composite used for the positive electrode and as-prepared composite was calcined at 600 °C and 800 °C. The morphological structures and pore-size distributions of MnYP-600HTT and MnYP-800HTT were characterized by means of XRD, SEM, EDAX, TEM, and BET. According to the BET specific surface-area evaluation, MnYP-600HTT and MnYP-800HTT were 1272.6 and 1388.1 m^2^/g, respectively. Total pore volumes were 0.627 and 0.687 cm^3^/g, which is beneficial for forming ion-transport channels in electrochemical reactions. The MnYP-600HTT electrode had a high specific capacity of 177.2 mAh/g at 20C, and the capacity retention was 64.8%. During the entire cycling, MnYP-600HTT had excellent cyclic stability in 500 cycles along with high efficiency. The robust design of the MnYP-600HTT and MnYP-800HTT cathode materials introduced in this work pave the way for designing next-generation supercapacitors operating at ultra-high C rates. The Mn-doped high surface of activated carbon had stable energy density and superior cycling stability that were demonstrated in supercapacitor systems.

## Introduction

The development of new technologies for the storage and production of electricity is a green way to prevent / reduce the environmental pollution. One of most suitable mechanism is supercapacitor, and it has the rapid charging/discharging, stable recycling performance with high rate-capability and low cost with environment friendliness^1–5^.

One way to change the performance of the electrodes in a capacitor is to develop a nano-sized active material with a high surface area with particular capacitance. Carbon-based materials have been used as high-capacity electrode materials for decades^6,7^. Some researchers synthesize carbon-based electrodes with some transition metals because of their high storage capability and unique reversibility^8–10^, but these kinds of materials still have some defects such as high price, inefficient electro-chemical stability and the block of the pores of carbon material that act as ion-transfer^11^. Surface activation is the most widely used method of improving the properties of activated carbon. In addition, the creation of artificial layer on the active-material exterior to prevent any side reaction mechanism between the electrode and electrolyte, if this attempt is successful the result is good long-term cycling performance.

Potassium permanganate is one of the nominated reagent for the surface activation reaction^12^. The role of KMnO_4_ is as a surface modifier and intermediate bridge or activator for ion transfer and it is oxidizing agent that enhances the exterior functional-groups of the active material^13^. RuO_2_^14^, NiO^15^, Co_3_O_4_^16,17^, MnO_2_^18^, NiCo_2_O_4_^19^, CoNiO_2_^20^, and Fe–V–O^21^ are used as cathode materials. The working potential of th cathode material is above 0 V vs. SCE and the anode material is below 0 V vs. SCE.

Mn-treated activated carbon and graphene were studied for the anode material in capacitor. Such as, Mn_3_O_4_/3D-graphene as positive electrode^22^, Mn-doped Fe_2_O_3_@rGO NPs as anode^23^, LiMn_2_O_4_@CNTs@graphene composite for cathode for aqueous hybrid capacitors ^24^ and Mn-doped ZnS nanosheets (ZnS:Mn NS) as the positive^25^. Mn-treated active material had high specific capacitance and excellent cycling profile.

In this regard, Mn-doped HSAC composites were used for the positive electrode and LaNi_5_ was used for the negative electrode. Mn-doped HSAC composites were calcined at 600 °C and 800 °C. As-prepared MnYP-600HTT and MnYP-800HTT cathode electrodes proved to have an exceptional electrochemical performance, with a specific surface area of 1272.6 and 1388.1 m^2^/g, a specific capacity of 294.7 mAh g^−1^ of the charging state, and 258.4 and 246.1 mAh g^−1^ of the discharging state in the Charge/Discharge profile after 10 cycles. A Mn-doped HSAC two-cathode electrode had good stability under a 500-cycle test. Those results are higher than those of pure pristine, which confirms that MnYP-600HTT and MnYP-800HTT electrodes are stable under long-time cycles. By comparing these three cycling performance curves, it can be seen that there is a process of activating capacity that happens during the initial cycles. This approach presented herein offers a promising route for the rational design of a new class of supercapacitors.

## Experiment part

### Preparation of Mn-doped high surface area Activated carbon (HSAC)

1 g of pure pristine diffused into as-prepared 1.5 M of HCl, HNO_3_ and H_2_SO_4_ acidic-solvent. The solution was mixed for 6 h and this was followed by a vaporization process. The mixture transferred into oven and dried at 110 °C for 8 h until it turns into a powder. Then acid-treated powder dispersed into 0.5 M concentrated sub-active reagent solution and stirred for 2 h at 80 °C to create a chemical bond between the sub-active material and activated carbon. Finally, the composite dried at 100 °C to obtain a powder and then calcined at 600 °C for 2 h for purification and to improve their surface properties.

### Anode manufacturing method

**Figure 1:**
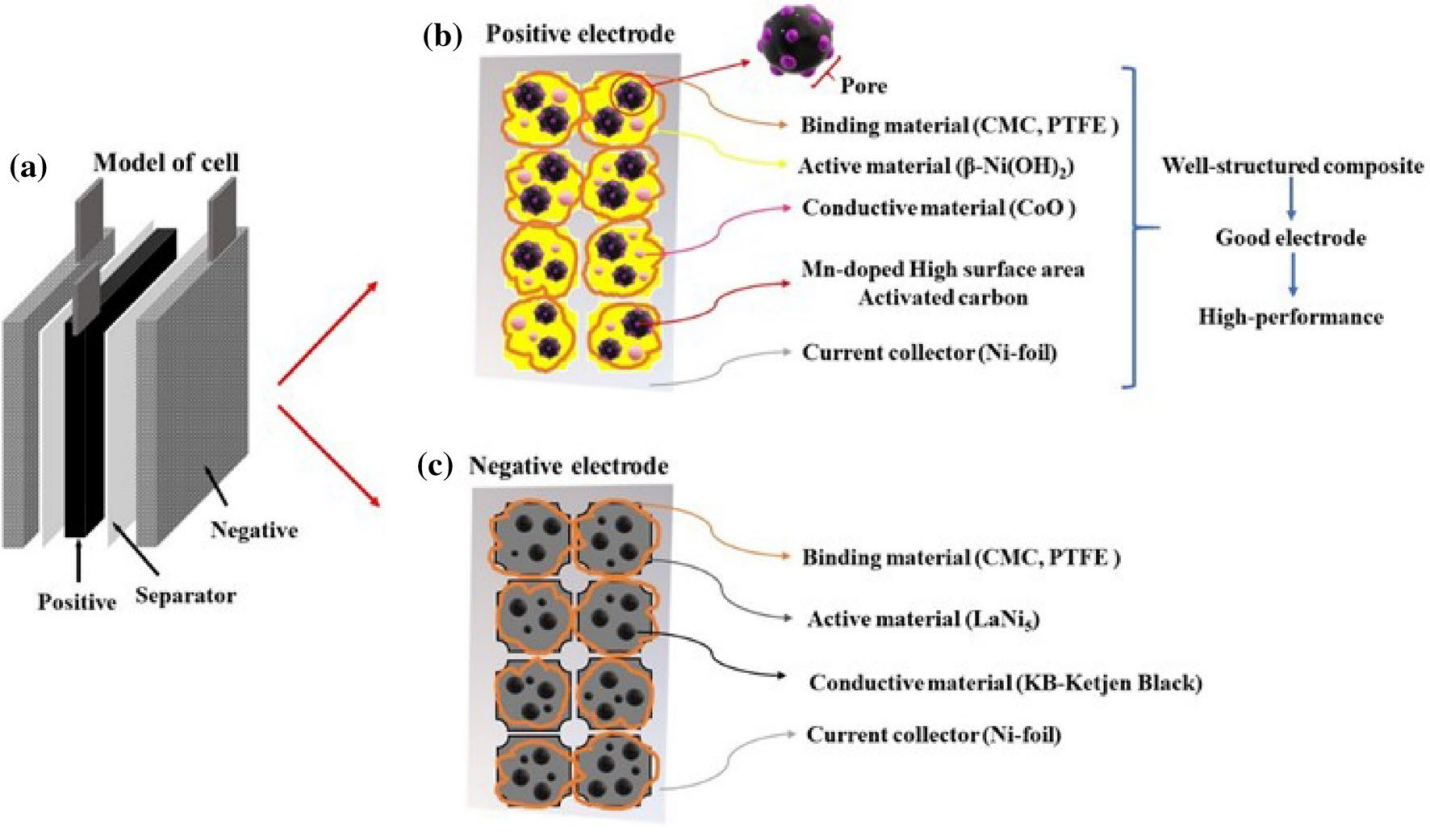
(**a**) Schematic illustration of the model of the Ni-hybrid capacitor, (**b**) detailed structure of the positive electrode, (**c**) detailed structure of the negative electrode.

A positive electrode was prepared using β-Ni(OH)_2_, a conductive material CoO (Alfa Aeasar), a binder CMC (Carboxymethyl cellulose), and PTFE (Polyetrafluouroethylene), and the electrode composition was 95:1:2:2 wt%. In the electrode manufacturing process, binders CMC (Carboxymethyl cellulose) and PTFE (Polyetrafluouroethylene) were dispersed in DI-water, and then β-Ni(OH)_2_ and CoO were added to prepare a slurry electrode using a mixer (Thinky mixer). The electrode slurry to which the activated carbon additive was applied was added in 10 wt% of the active material to prepare a slurry electrode. The slurry electrode thus prepared was coated on a Ni-foam type current collector by applying a dip coating process, dried in a 100 °C oven, and pressed to a thickness of 300 μm using a roll press equipment. The schematic illustration of the anode was displayed in Fig. 1.

### Cathode manufacturing method

A negative electrode was manufactured using LaNi5, a conductive material KB (Ketjen Black), a binder CMC (Carboxymethyl cellulose), and PTFE (Polyetrafluouroethylene), and the electrode composition was 93:4:2:1 wt%. In the electrode manufacturing process, binders CMC (Carboxymethyl cellulose) and PTFE (Polyetrafluouroethylene) were dispersed in DI-water, and LaNi_5_ and KB were added to prepare a slurry electrode using a mixer (Thinky mixer). The slurry electrode thus prepared was coated on a Ni-foam type current collector by applying a dip coating process, dried in a 100 °C oven, and pressed to a thickness of 250 μm using a roll press equipment. The schematic illustration of the cathode was displayed in Fig. 1.

### Cell manufacturing process

We prepared an electrode with a capacity ratio of 1:2.5 of the positive electrode and the negative electrode. A separator was placed between the two negative electrodes, and the positive electrode was placed in the middle to prepare a cell. We impregnated it with 10 g of 6 M KOH and 0.3 M LiOH in distilled deionized (DDI) water and kept it under vacuum for 12 h, followed by a cell test.

### Characterization

The crystalline structure was examined by X-ray diffraction instrument (SHIMADZU XRD-6000) with 1°/min scan rate. The morphology of the KMnO_4_-HSACs were analysed using SEM/TEM and HRTEM (JSM-5600 JEOL, Japan) (TEM, Hitachi HT7700, operated at 100 kV). The presence of the elements was analysed via EDX analysis conducted into SEM. The surface chemical state of material was analysed via X-ray photoelectron spectra (KRATOS AXIS SUPRA).

### Electrochemical test

To form a nickel hybrid capacitor cell with electrolyte aging completed, it was charged with a constant current of 0.1C and discharged to 1.0 V at a constant current of 0.1C, which was done for 10 cycles. After cell formation was complete, we measured a Nyquist plot at a frequency range of 1–1 MHz and an amplitude of 10 mV for EIS (Electrochemical Impedance Spectroscopy) analysis. To evaluate the output characteristics according to the current of the cell, we did a rate capability test in the current range of 0.2–30 C, with the cell being charged and discharged up to 500 cycles at a constant current of 1C.

## Result and discussion

### X-ray diffraction studies and morphological characterization

**Figure 2:**
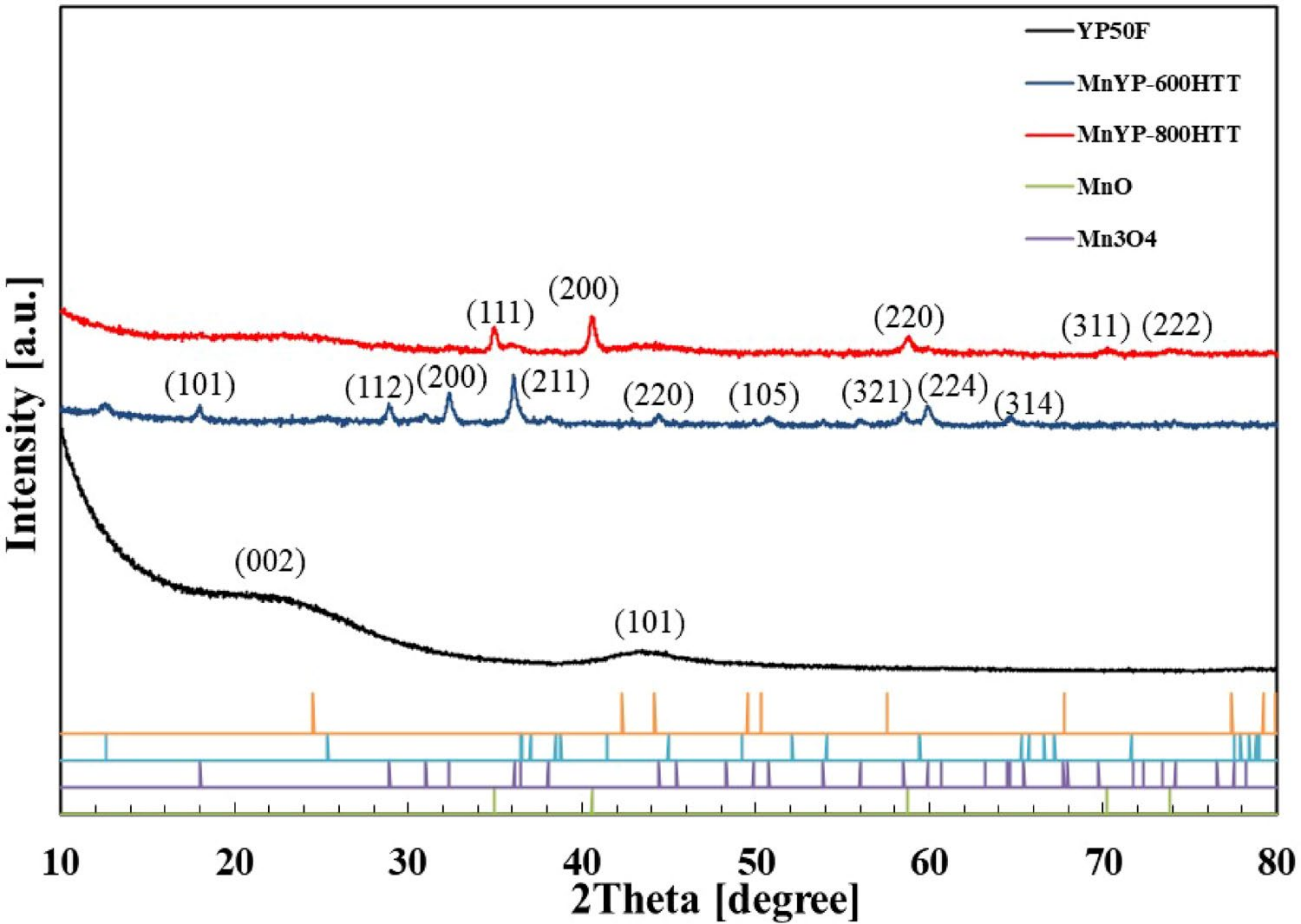
XRD pattern of Mn-doped MnYP-600HTT and MnYP-800HTT.

The XRD pattern of Mn-doped activated carbon after heat treatment at 600 and 800 °C is shown in Fig. 2. We ascribed the peaks to the presence of MnO in Mn-YP800HTT. Diffraction peaks at 2*θ* of 34.9, 40.6, 58.8, 70.3, and 73.8 were ascribed to the crystalline planes (111), (200), (220), (311), and (222), respectively, of MnO (JCPDS No. 89-4835) to confirm the presence of Mn on the activated carbon surface. For Mn-YP600HTT, all peaks showed the presence of Mn_3_O_4_. The diffraction peaks were ascribed to the crystalline planes (112), (103), (211), (004), (220), and (224) of Mn_3_O_4_ (JCPDS Card No.00-001-1127). According to the XRD result, the temperature difference can affect the crystal structure of Mn.

**Figure 3:**
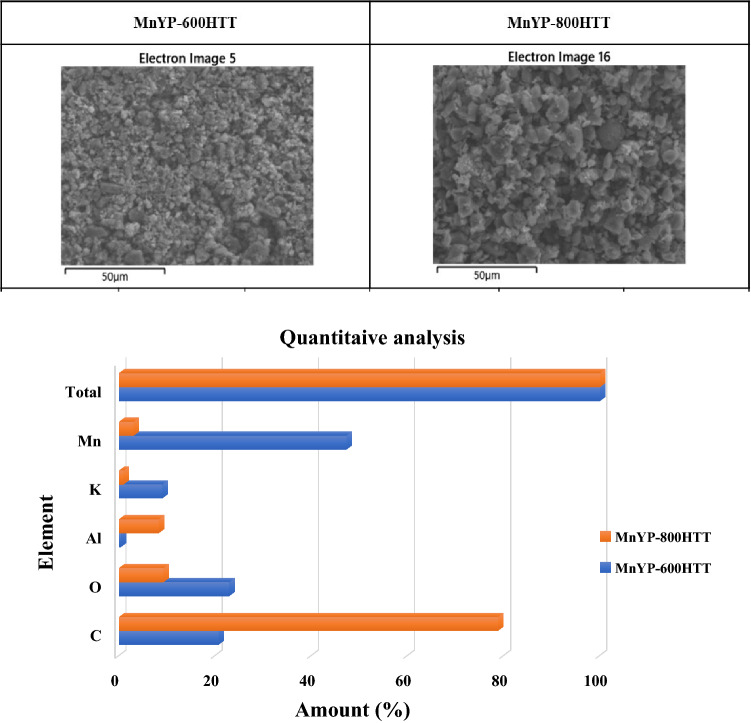
The quantitative analysis results.

The quantitative as well as qualitative number of the main elements in the catalyst were detected via EDX analysis, which also revealed each element’s characteristic peak (sharp K_α_ and K_β_). Figure 3 displays the micro-analysis results of all as-synthesized samples; it shows the presence of the main elements. K, Mn, and O come from the doping reagent, whereas C comes from the activated carbon. According to the EDAX analysis, a small amount of Al and Cl were detected. In addition, the amount of Mn was not the same, because of temperature dependency.

**Figure 4:**
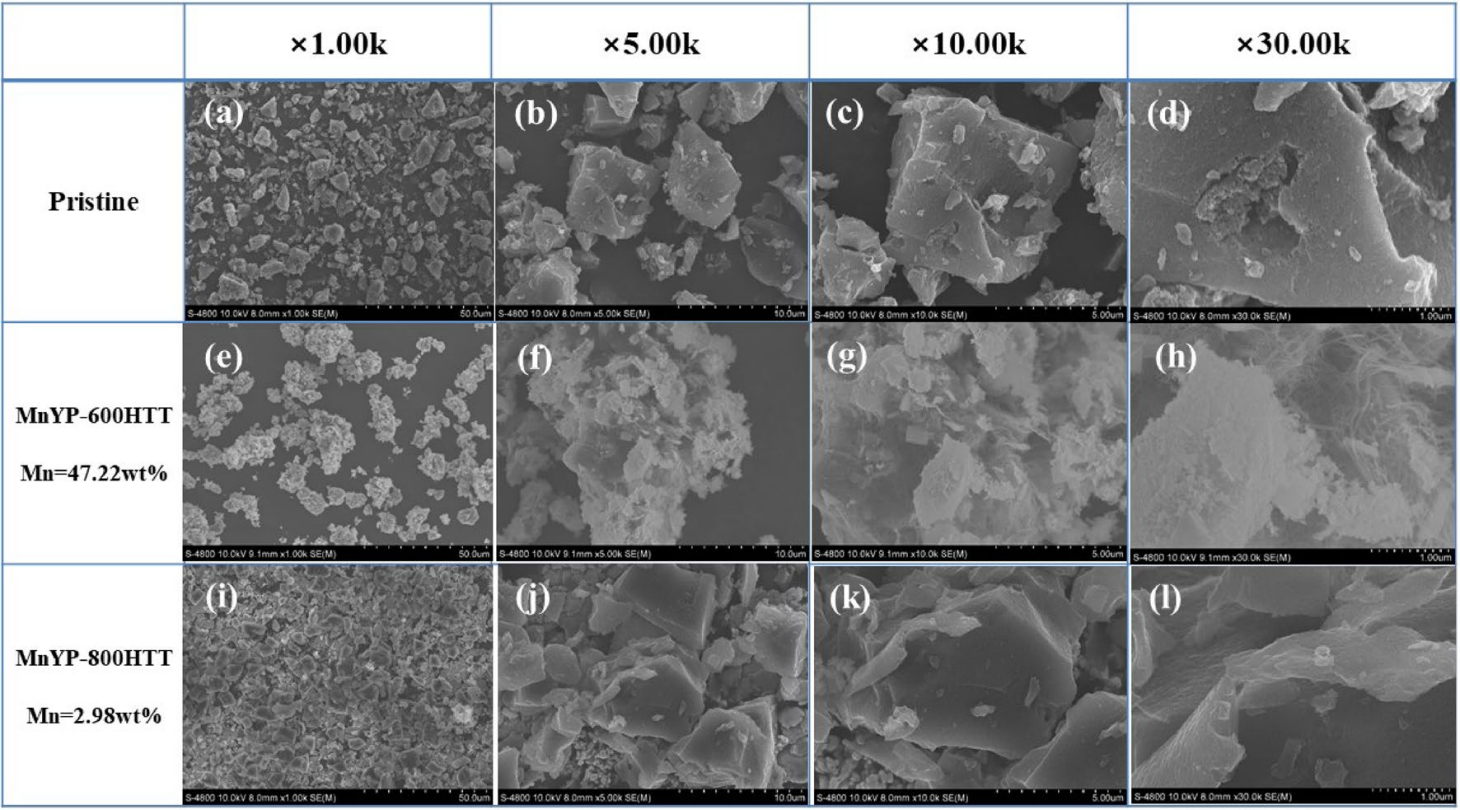
SEM images of Pristine, Mn-doped MnYP-600HTT and MnYP-800HTT.

We investigated the surface morphology with SEM. Figure 4 shows the SEM analysis results of the Mn-doped high surface area of activated carbon after 600 °C and 800 °C calcination. The pure pristine had several structures of particles, as shown in Fig. 4a–d. From the image, it can be seen that the Mn particles fully covered the surface of activated carbon in MnYP-600HTT. The morphology of MnYP-600HTT was web-shaped, as shown in Fig. 4e–h. For MnYP-800HTT, the surface of each particle was smooth and covered by Mn. All particles are gathered in Fig. 4i–l. The amount of Mn was not the same in each prepared sample, which can affect the surface and property of a composite material. The main role of the large surface area of the pristine is to provide a sufficient area of the nanocomposite that can be grown on the surface during synthesis. In addition, the graphene made a chemical bond with precursor reagents by means of oxygen-containing functional groups. Furthermore, the results of SEM showed the distinction between the Mn-doped HSAC composite under the 600 °C and that under 800 °C.

**Figure 5:**
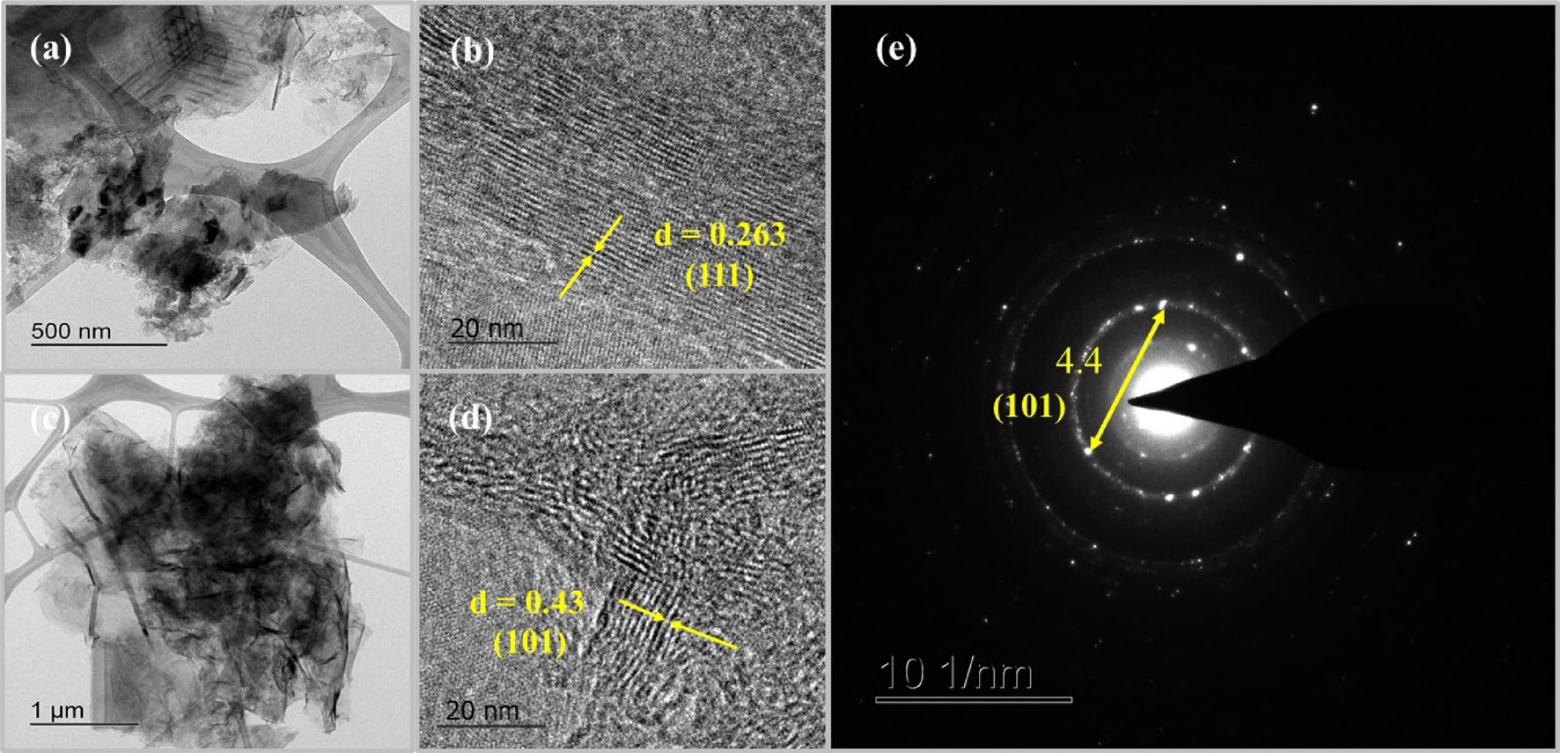
(**a**–**d**) TEM and HRTEM images of Pristine, Mn-doped MnYP-600HTT and MnYP-800HTT, (**e**) SAED pattern of Mn-doped HSAC.

The atomic structure and information of crystallographic structure of nanocomposite was analyzed via HRTEM. Figure 5a,c shows the TEM images of Mn-doped HSAC which exhibited that Mn-particles had irregular shape and all materials is enveloped in high surface area of activated carbon, resulting in a good composite. Figure 5b,d indicated the lattice fringes of Mn-doped HSAC composite under the 600 °C and that under 800 °C. The lattice fringes with d-spacing of Mn-doped HSAC was 0.263 and 0.43 nm and these are corresponding to the (111) and (101) crystal planes of MnO and Mn_3_O_4_, respectively. The selected area electron diffraction (SAED) was used to fully reconstruct the reciprocal space. Figure 5e shows the (101) zone axis and width was 4.4 nm which can be indexed on the basis of a Mn_3_O_4_.

**Figure 6:**
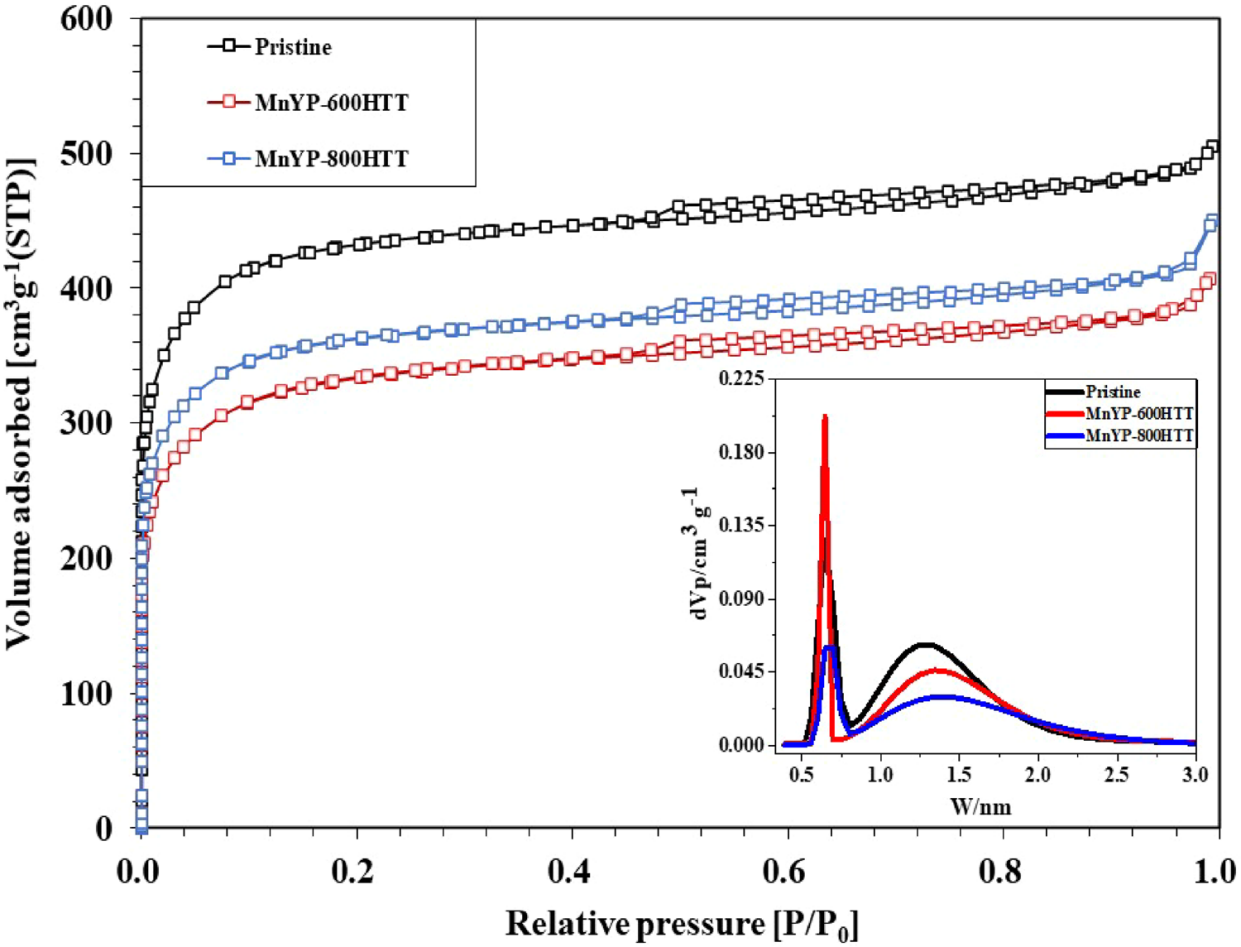
Nitrogen adsorption–desorption isotherms of Pristine, Mn-doped MnYP-600HTT and MnYP-800HTT.

The specific surface area, pore diameter, and pore volume affect the electrochemical properties. Figure 6 shows the N_2_-adsorption-desoprtion isotherm of MnYP-600HTT and MnYP-800HTT composites. BET and BJH method used for the analysis of specific surface area and pore-size distribution. The calculation results were summarized in Table 1. The relative pressure is (0.5–1.0) P/P0, as shown in Fig. 6. According from Fig. 6, relation between pore-distribution and isotherm belonged to the H1, this result can confirm the all composites has slender pore-size. The isotherm type IV is characteristic of mesoporous/micropores adsorbents. The specific surface area of pure pristine was 1676.1 m^2^g^-1^, and the average pore diameter was 1.85 nm. After the synthesis, the specific surface area and mean pore diameter were increased in MnYP-800HTT. According to the calculated T-plot, the total surface area was high in pristine. After Mn-dope and calcination, the surface area decreased, but MnYP-800HTT still had a larger surface area than did MnYP-600HTT. The total specific surface area of MnYP-600HTT was 1412.6 m^2^/g, whereas MnYP-800HTT had 1578.6 m^2^/g. Micropore volume was decreased from 0.693 to 0.567 cm^3^/g, and mesopore volume increased from 0.084 to 0.120 cm^3^/g in the MnYP-800HTT sample, which is beneficial for forming ion-transport channels in electrochemical reactions.

**Table 1:** Nitrogen adsorption–desorption isotherms of Pristine, Mn-doped MnYP-600HTT and MnYP-800HTT.

Mn-doped A.C. additives	Pore structure parameter								
	BET			T-plot					
	Surface area (m^2^/g)	Total pore volume (cm^3^/g)	Mean pore diameter (nm)	Total surface area (m^2^/g)	Micropore surface area (m^2^/g)	External surface (m^2^/g)	Micropore volume (cm^3^/g)	Mesopore volume (cm^3^/g)	Micropore vol. percent (%)
Pristine	1676.1	0.776	1.85	1898.4	1639.4	36.7	0.693	0.084	89
MnYP-600HTT	1272.6	0.627	1.97	1412.6	1236.6	36.0	0.534	0.093	85
MnYP-800HTT	1388.1	0.687	1.98	1578.6	1344.4	43.7	0.567	0.120	83

**Figure 7:**
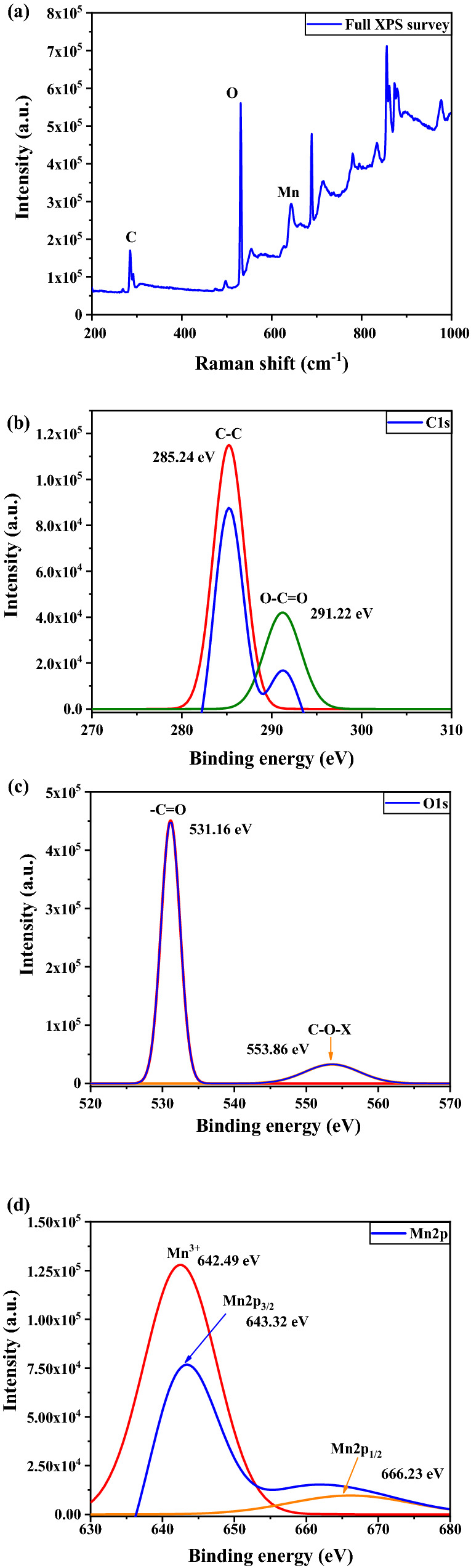
Survey XPS spectrum (**a**) and high-resolution XPS spectra of (**b**) C1s, (**c**) O1s, (**d**) Mn2p.

To explore the detailed surface chemical state of the elements and interactions between Mn and HSAC, we did an XPS analysis of Mn-doped HSAC. The binding energies depend on the chemical structure and elemental formation of the sample. Figure 7a presents the XPS survey spectrum, which shows the existence of C1s, O1s, Mn, and K, thus confirming the successful formation of Mn-doped activated carbon. Figure 7b shows the C1s XPS spectrum, which consists of some characteristic peaks, such as carbonyl carbon (C=O, 288.4–288.6 eV), aliphatic (C–C, 284.4–285.9 eV) and carboxylate carbon (O–C=O, 289.3 eV)^26^. These peaks corresponded to the state of carbon with carbon bonded by a single bond (C–C) and the state of carbon linked by a single bond to an oxygen-containing group (C–O(O)). The C1s XPS shows five notable peaks corresponding to non-oxygenated carbon (285.24 eV) and carboxylate group (291.22 eV)^27^. Figure 7c shows the O1s spectra on graphene and Mn. The peak location relates to the functional group, such as, the O1s peak located at over 531 eV region express the metal-oxide group, if it is below 531 eV can express the carbonyl or hydroxide group^28^. In the XPS spectrum of O1s, the broad peak was located at 531.16 eV, which indicated the carbonyl group. In addition, The second-peak which located at 553.86 eV expresses the chemical interaction between metal (X) and oxygen. The electronic configuration of Mn2p is shown in two peaks, which correspond to the mixed valence state, as shown in Fig. 7d. Mn 2p_1/2_ is located at the 666.23 eV binding energy region, and the Mn 2p_3/2_ peak can be deconvoluted into two peaks of 30.34% Mn^3+^ (643.32 eV), and 29.54% Mn^4+^ (647.28 eV), corresponding to the spin–orbit peaks of Mn with mixed valence states of + 3 and + 4, respectively^29^. The binding energy shifts indicate the strong bond-interaction and charge transfer between the Mn and HSAC.

### Electrochemical evaluation

**Figure 8:**
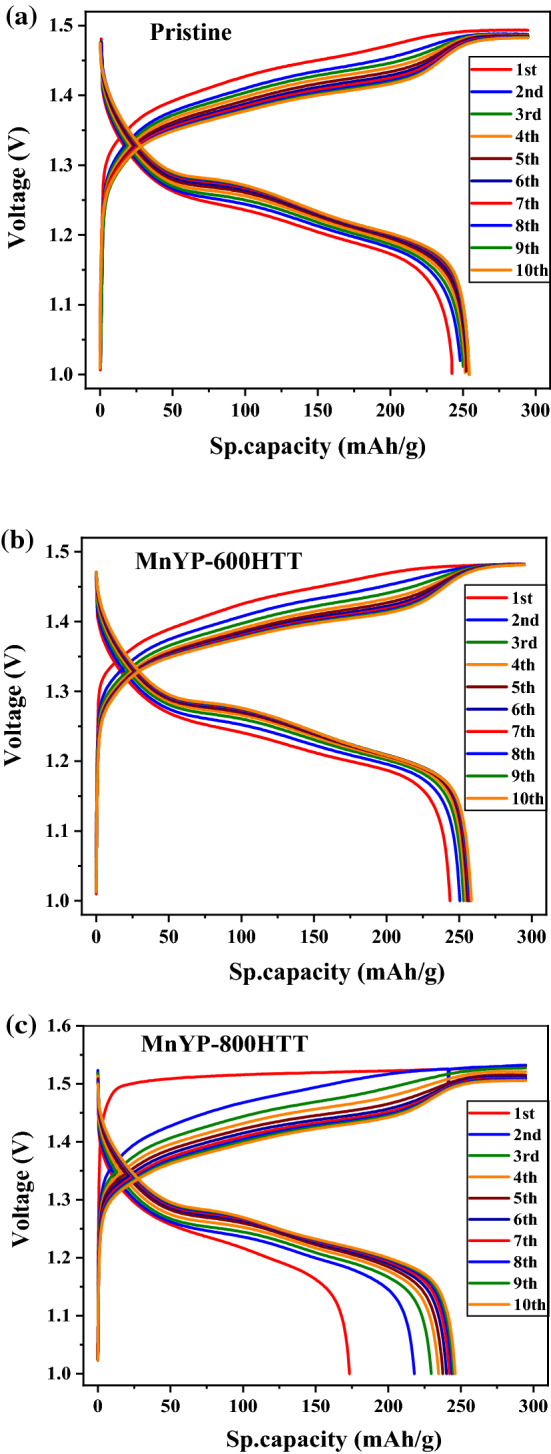
The charge–discharge profiles of Pristine, Mn-doped MnYP-600HTT, MnYP-800HTT.

Figure 8 presents the charge–discharge profiles of pure pristine and MnYP-800HTT. The negative electrode was LaNi_5_ and the as-prepared samples were the positive electrode under the applied current. The charge–discharge profiles experiment was Super charging at 0.01C, 0.02C, 0.05C × 5 h (Cut-off time); Super discharge at 0.05C to 1.0 V (Cut-off voltage); Charging at 0.1C × 12 h (120% charging); and discharging at 0.1C MnYP-600HTT, 1.0 V (Cut-off voltage). During a ten-cycling test, all samples had high capacity, and MnYP-800HTT showed quite a different charge–discharge profile because of the amount of Mn and specific surface area. According to the EDAX analysis, the amount of Mn in MnYP-800HTT was 2.98% and specific surface area and total surface area were higher than in MnYP-600HTT, which was close to pure pristine. Moreover, the MnYP-600HTT electrode has an obvious charging platform, and the charging characteristics of the MnYP-600HTT and MnYP-800HTT electrodes are more consistent with similar capacitance characteristics.

**Figure 9:**
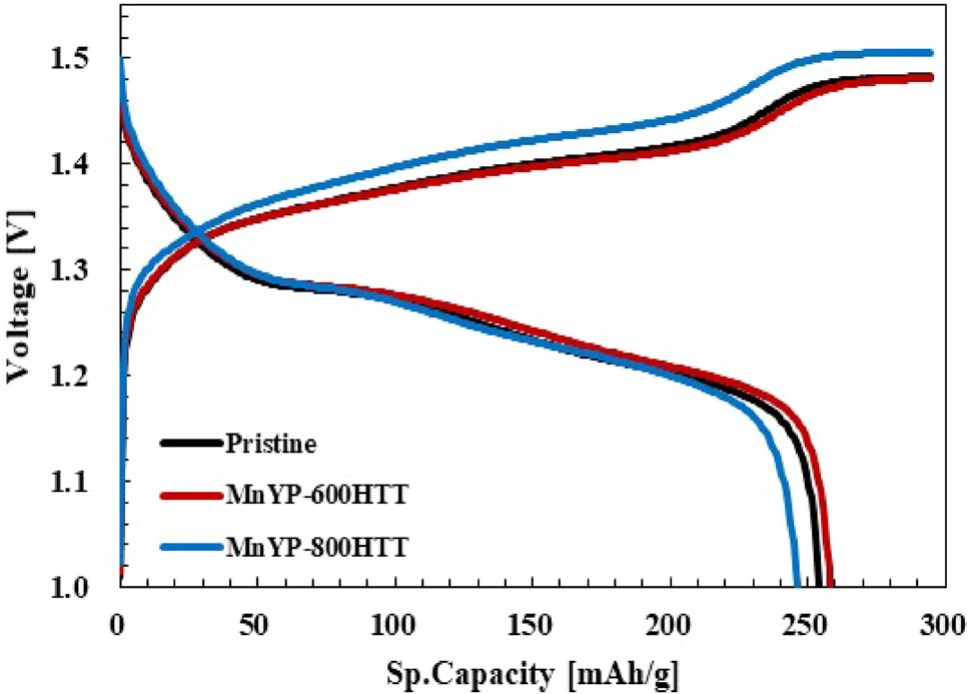
The charge–discharge profiles of Pristine, Mn-doped MnYP-600HTT and MnYP-800HTT after 10 cycles.

Figure 9 shows the charge–discharge profiles of the hybrid device composed with LaNi_5_ as the negative electrode and the pristine, MnYP-600HTT, and MnYP-800HTT as the positive electrode under an applied current of state. The experiment was a charge/discharge test (10 cycles): Charging 0.2C × 6 h (cut-off time, 120% charging); Discharge 0.2C, 1.0 V (Cut-off voltage). The specific capacity of the positive electrode is calculated in Fig. 9. In the charge state, the amounts of capacity [mAh] of each positive electrode were 94.9 mAh in Pristine, 93.4 mAh in MnYP-600HTT, and 80.0 mAh in MnYP-800HTT. In the charged state, the capacities of Pristine, MnYP-600HTT, and MnYP-800HTT were 94.9, 93.4, and 80.8 mAh. The capacities were 81.9, 81.9, and 66.8 mAh in the discharged state. The specific capacities of Pristine, MnYP-600HTT, and MnYP-800HTT were equal to each other, at around 294.7 mAh/g. Specific capacities were 254.5, 258.4, and 246.1 mAh/g in the discharged state. The ultra-specific capacity was successfully demonstrated in MnYP-600HTT. The charging and discharging capacity and specific capacity of MnYP-800HTT were less than those of the other two samples. A table for comparison between the Mn-doped carbon materials for supercapacitors is given in Table 2.

**Table 2:** A table for comparison between Mn-doped carbon materials for supercapacitor.

Positive	Negative	Charge		Discharge	
		Capacity (mAh)	Sp. capacity (mAh/g)	Capacity (mAh)	Sp. capacity (mAh/g)
Pristine	LaNi_5_	94.9	294.7	81.9	254.5
MnYP-600HTT	LaNi_5_	93.4	294.7	81.9	258.4
MnYP-800HTT	LaNi_5_	80.0	294.7	66.8	246.1

**Figure 10:**
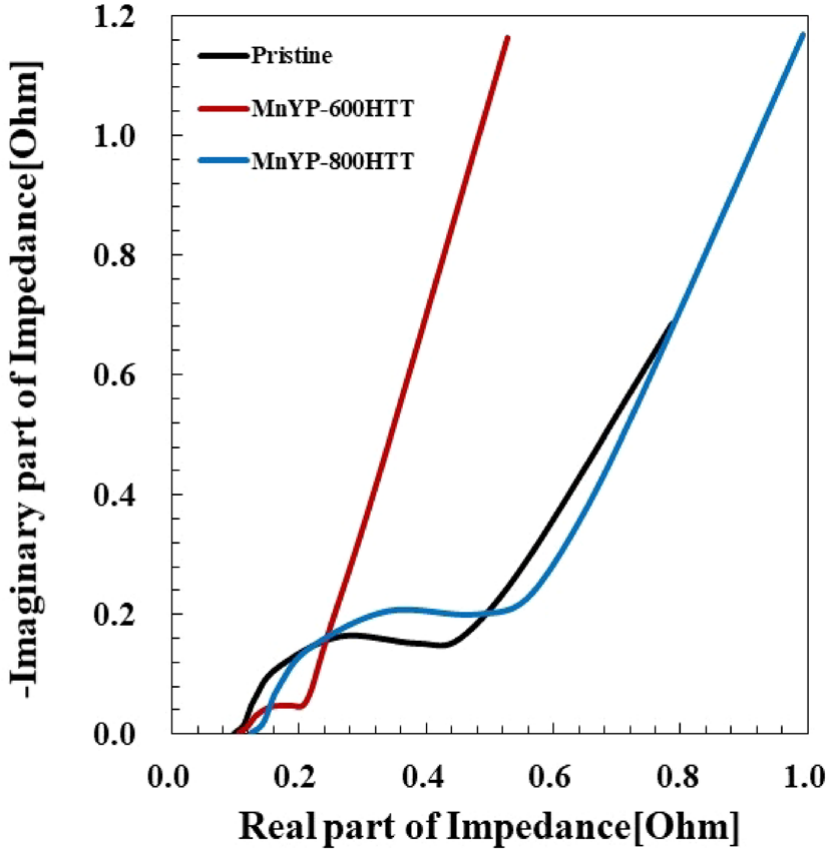
Nyquist plot of Pristine, Mn-doped MnYP-600HTT and MnYP-800HTT.

Figure 10 shows the Nyquist plots of the electrodes, displaying two depressed semicircles within the frequency range of 1–1 MHz and 10 mv of amplitude. Theoretically, the first semicircle (under high frequency) may be related to the Mn + displacement passing through the electrode and the next semicircle related to the charge transfer between electrode/electrolyte. Lastly the Mn ions may diffuse on the electrodes under the low frequency condition. The real axis state and semicircle profile can relate to the resistivity of the electrolyte, interaction between active material and current-collector, charge transfer property. The deviation of the pristine and MnYP-800HTT was around 40%MnYP-600HTT was 20% in the 0.1–1.0 Ω impedance range. As can be shown in Fig. 10, we measured *R*s of 0.70, 1.16, and 1.17 Ω for pure pristine, Mn-doped MnYP-600HTT, and Mn-doped MnYP-800HTT electrodes, respectively. The Mn-doped MnYP-600HTT nanocomposite shows lower *R*s than does the Mn-doped MnYP-800HTT nanocomposite, attributed to the doping effect by Mn in the MnYP-600HTT and MnYP-800HTT nanocomposite. Lower resistance confirmed that the sample has a high conductive property. Comparing the EIS of the three cases, MnYP-600HTT and MnYP-800HTT had a significant slope on the Y axis in the low-frequency region due to the easy absorption/reception of electrode ions. Furthermore, in the low-frequency region, the EIS of Mn-doped MnYP-600HTT is even more inclined to the imaginary axis than is the Mn-doped MnYP-800HTT, indicating that Mn doping in the electrodes promotes the formation of a more effective channel, which is more conducive for the ion diffusion.

**Figure 11:**
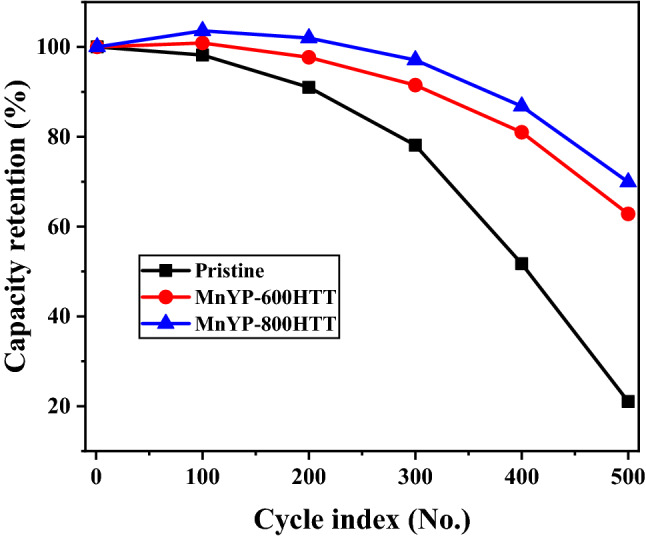
Cycling test of Pristine, Mn-doped MnYP-600HTT and MnYP-800HTT under 500 cycles.

We tested the cycling stability of pure pristine, Mn-doped MnYP-600HTT, and Mn-doped MnYP-800HTT electrodes by using GCD measurements at 1 V for 500 cycles, as shown in Fig. 11. The values of capacitance retention for pure pristine, Mn-doped MnYP-600HTT, and Mn-doped MnYP-800HTT electrodes were 100% of the maximum capacitance at the first cycle. After the 500th cycling test, the Mn-doped MnYP-600HTT and Mn-doped MnYP-800HTT electrodes were at 62.8 and 69.9% of the 52.3 and 43.9 mAh capacity. Those results are higher than for pure pristine, which confirms that Mn-doped MnYP-600HTT and MnYP-800HTT electrodes are stable under long-time cycles.

The activation process happens in the initial recycling step and the activation time depend on the active material. Mn-doped electrode required a long activation time due to recrystallization. Above mentioned factor affects the decay-time of MnYP-600HTT and MnYP-800HTT in the charge/discharge cycles. Clearly, Mn-doped MnYP-600HTT and MnYP-800HTT can achieve both relatively high specific capacity and good cycling behavior, simultaneously. The specific capacity of Mn-doped MnYP-600HTT was 262.8 mAh/g, and Mn-doped MnYP-800HTT had 231.1 mAh/g at the 1st cycle. After the 500th cycle, the specific capacities were decreased to 165.0 and 161.6 mAh/g, respectively. The capacity, specific capacitance, and capacity retention (%) of all samples are summarized in Table 3. More specifically, Mn-doped HSAC materials exhibited a high specific capacitance, and the loss of specific capacities was around 37.21% and 30.07% in each Mn-doped sample. In addition to such a high mass loading, high specific capacity and good cycle performance were also achieved, indicating that our method is feasible for practical application of the positive electrode materials in a supercapacitor.

**Table 3:** Cycling test result of Pristine, Mn-doped MnYP-600HTT and MnYP-800HTT under 500 cycles.

Mn-doped A.C. additives	Cycle index (no.)					
	1st	100th	200th	300th	400th	500th
**Pristine**						
Capacity (mAh)	82.1	80.6	74.7	64.1	42.4	17.3
Sp.Capacity (mAh/g)	261.1	256.4	237.5	204.0	134.9	54.9
Capacity retention (%)	100.0	98.2	91.0	78.1	51.7	21.0
**MnYP-600HTT**						
Capacity (mAh)	83.2	84.0	81.3	76.2	67.4	52.3
Sp.Capacity (mAh/g)	262.8	265.2	256.7	240.5	212.8	165.0
Capacity retention (%)	100.0	100.9	97.7	91.5	81.0	62.8
**MnYP-800HTT**						
Capacity (mAh)	62.7	64.9	64.0	60.9	54.4	43.9
Sp.Capacity (mAh/g)	231.1	239.4	235.8	224.5	200.7	161.6
Capacity retention (%)	100.0	103.6	102.0	97.1	86.8	69.9

**Figure 12:**
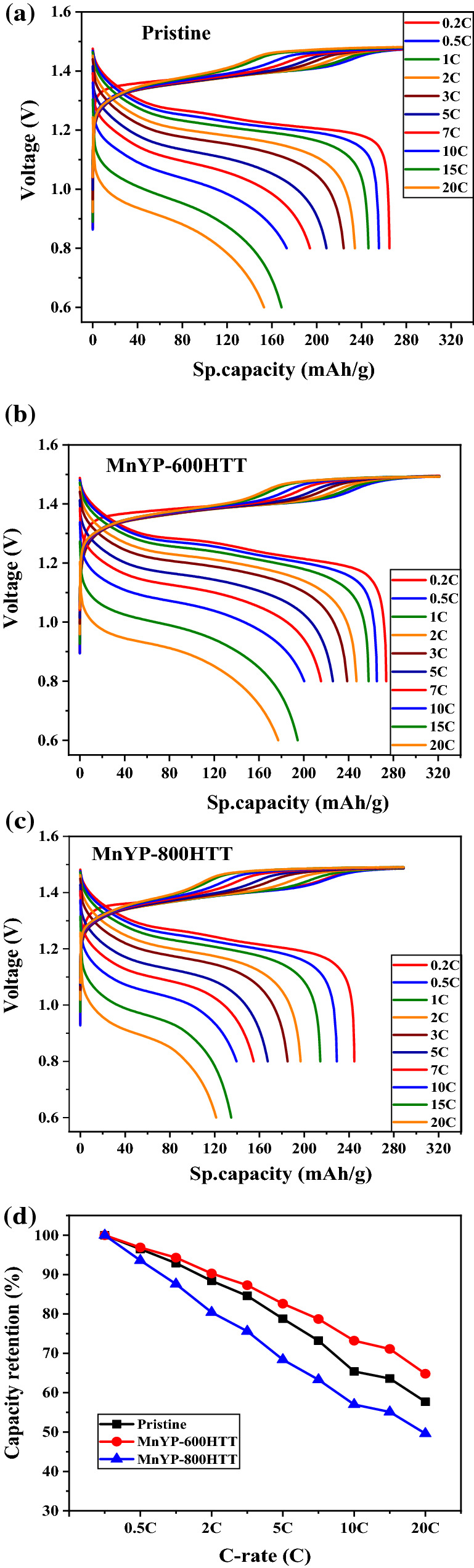
Rate capability test of Pristine, Mn-doped MnYP-600HTT and MnYP-800HTT under different C.

The rate capability performance was conducted for the Mn-doped HSAC cathode electrode. As shown in Fig. 12, the Mn-doped MnYP-600HTT cathode electrodes outperformed the pure Pristine electrode at various cycling conditions. The peaks were obtained in the 0.6–1.5 voltage region. The specific capacity of the Mn-doped HSAC cathode electrode is calculated in Fig. 12. The specific capacity of the pure Pristine was 265.0 mAh/g at 0.2C and decreased to 152.9 mAh/g at 20C. For the MnYP-600HTT, MnYP-800HTT had 273.7 mAh/g and 244.7 mAh/g. This result decreased to 177.2 mAh/g and 121.3 mAh/g at 20C. According to the above result, the Mn-doped MnYP-600HTT cathode had high capacity because of the amount of Mn-doping. The capacity retention of MnYP-600HTT decreased from 100 to 64.8%. The retention of MnYP-800HTT decreased from 100 to 49.6%. All results are summarized in Table 4.

**Table 4:** Rate capability property of Pristine, Mn-doped MnYP-600HTT and MnYP-800HTT under different C.

Mn-doped A.C. additives	C-rate (C)									
	0.2C	0.5C	1C	2C	3C	5C	7C	10C	15C	20C
**Pristine**										
Sp. capacity (mAh/g)	265.0	255.6	246.2	234.1	224.2	208.7	194.0	173.2	168.6	152.9
Capacity retention (%)	100.0	96.5	92.9	88.4	84.6	78.8	73.2	65.4	63.6	57.7
**MnYP-600HTT**										
Sp. capacity (mAh/g)	273.6	265.2	257.9	247.0	238.7	225.9	215.4	200.3	194.5	177.2
Capacity retention (%)	100.0	96.9	94.3	90.3	87.3	82.6	78.7	73.2	71.1	64.8
**MnYP-800HTT**										
Sp. capacity (mAh/g)	244.7	229.1	214.3	196.7	185.1	167.4	154.8	139.5	134.8	121.3
Capacity retention (%)	100.0	93.6	87.6	80.4	75.6	68.4	63.3	57.0	55.1	49.6

All the above results confirmed that Mn-doped MnYP-600HTT electrode had a high rate capability and electrochemical property. The prominent rate capability of the Mn-doped MnYP-600HTT and MnYP-00HTT electrode can be ascribed to the unique structure, in which HSAC formed connections with Mn. The structure of the active material can upgrade the contact area between electrolyte and electrode surface, redox reaction rate. As a result, the ion-transfer performance of active-material increase in the electrochemical analysis. The total electrochemical results confirmed that a certain amount of Mn-doping under a correct calcination process can enhance the electrochemical property of HSAC materials. Furthermore, our mentioned method and results confirmed that Mn with an ultra-high surface area of activated carbon exhibited a good electrochemical property and upgraded the class of supercapacitors. Recently, some research papers reported Mn-doped graphene oxide and activated carbon for the supercapacitor, such as Mn-doped Fe_2_O_3_@rGO hollow core–shell spheres, MnO nanoparticles with fluorine-doped carbon^30^, V_2_O_5_/polyindole (V_2_O_5_/PIn) decorated onto the activated carbon^31^. Other supercapacitor materials have been studied in the field of energy storage devices, such as 3D Na_2_Ti_3_O_7_ nanosheet arrays/carbon textiles anodes joined sodium‐Ion pseudocapacitors^32^ NiS_2_ nanosheets on porous carbon microtubes^33^. The Mn-doped Fe_2_O_3_@rGO hollow core–shell spheres increased the active regions of redox reactions, where remarkable electrochemical properties with a specific capacity of 285 mAh g^−1^ after the 1000th cycling test. The MnO nanoparticles with fluorine-doped carbon had 40 mAh g^−1^ after 200 cycles.

Above noted all capacitor material showed good conductivity, high energy and high safety. According to the above-mentioned articles, the specific capacitance of each sample was less than that of our prepared Mn-doped high surface area of activated carbon. Furthermore, the charge-transfer properties of the above samples had much less than the Mn-doped HSAC because of the big semicircle in the Nyquist plot. The Mn-doped high surface of activated carbon had stable energy density and superior cycling stability that were demonstrated in our supercapacitor system.

## Conclusion

In summary, the positive electrode was prepared by means of the Mn-doped high surface area of activated carbon (HSAC), and LaNi_5_ was used for the negative electrode. The prominent rate capability of the Mn-doped MnYP-600HTT and MnYP-00HTT electrode can be ascribed to the unique structure, in which HSAC formed connections with Mn. The special-structured active material increased the redox-reaction rate, contact area between electrolyte and electrode, it can improve the electrochemical performance of the material. Clearly, Mn-doped MnYP-600HTT and MnYP-800HTT can achieve both relatively high specific capacity and good cycling behavior, simultaneously. The specific capacity of Mn-doped MnYP-600HTT was 262.8 mAh/g, and Mn-doped MnYP-800HTT had 231.1 mAh/g at the 1st cycle. After the 500th cycle, the specific capacities were decreased to 165.0 and 161.6 mAh/g, respectively. The specific surface area of MnYP-800HTT was higher than that of MnYP-600HTT, and the Mn amount in MnYP-600HTT was 47.22%, and in MnYP-800HTT was 2.98%. The Mn-amount can affect the surface of HSAC and change the electrochemical property. The Mn-doped high surface of activated carbon had stable energy density and superior cycling stability as were demonstrated in our supercapacitor system.

## References

[1] Kang K, Ceder G (2009). Battery materials for ultrafast charging and discharging. Nature.

[2] Yang CC, Sun WC, Kumar A, Pattanayak B, Tseng TY (2019). Templating synthesis of nickel cobaltite nanoflakes and their nanocomposites for making high-performance symmetric supercapacitors. Mater. Today Energy.

[3] Simon, P., Gogotsi, Y. Materials for electrochemical capacitors, nanoscience and technology: A collection of reviews from nature journals. *World Sci.* 320–329 (2010).

[4] Jiang W, Yu D, Zhang Q, Goh K, Wei L, Yong Y, Jiang R, Wei J, Chen Y (2015). Ternary hybrids of amorphous nickel hydroxide–carbon nanotube-conducting polymer for supercapacitors with high energy density, excellent rate capability, and long cycle life. Adv. Funct. Mater..

[5] He G, Ling M, Han X, El Amaiem DIA, Shao Y, Li Y, Li W, Ji S, Li B, Lu Y (2017). Self-standing electrodes with core-shell structures for high-performance supercapacitors. Energy Stor. Mater..

[6] Zhou SZ, Zhou GY, Jiang SH, Fan PC, Hou HQ (2017). Flexible and refractory tantalum carbide-carbon electro spun nanofibers with high modulus and electric conductivity. Mater. Lett..

[7] Chen D, Hu X, Huang Y, Qian Y, Li D (2019). Facile fabrication of nanoporous BCN with excellent charge/discharge cycle stability for high-performance supercapacitors. Mater. Lett..

[8] Wang YC, Zhou T, Jiang K, Da PM, Peng Z, Tang J, Kong B, Cai WB, Yang ZQ, Zheng GF (2014). Reduced mesoporous Co3O4 nanowires as efficient water oxidation electrocatalysts and supercapacitor electrodes. Adv. Energy. Mater..

[9] Yu MH, Zeng Y, Han Y, Cheng XY, Zhao WX, Liang CL, Tong YX, Tang HL, Lu XH (2015). Valence-optimized vanadium oxide supercapacitor electrodes exhibit ultrahigh capacitance and super-long cyclic durability of 100,000 cycles. Adv. Funct. Mater..

[10] Ji HM, Liu XL, Liu ZJ, Yan B, Chen L, Xie YF, Liu C, Hou WH, Yang G (2015). In situ preparation of sandwich MoO3/C hybrid nanostructures for high-rate and ultralonglife supercapacitors. Adv. Funct. Mater..

[11] Ko WY, Liu YC, Lai JY, Chuang CC, Lin KJ (2018). The effect of EV aggregators with time-varying delays on the stability of a load frequency control system. ACS Sustain. Chem. Eng..

[12] Cho Y, Oh P, Cho J (2013). A new type of protective surface layer for high-capacity Ni-based cathode materials: nano scaled surface pillaring layer. Nano Lett..

[13] Sun YK, Lee DJ, Lee YJ, Chen Z, Myung ST (2013). Cobalt-free nickel rich layered oxide cathodes for lithium-ion batteries. ACS Appl. Mater. Interfaces.

[14] Warren R, Sammoura F, Tounsi F, Sanghadasa M, Lin L (2015). Highly active ruthenium oxide coating via ALD and electrochemical activation in supercapacitor applications. J. Mater. Chem. A.

[15] Kim SI, Lee JS, Ahn HJ, Song HK, Jang JH (2013). Facile route to an efficient NiO supercapacitor with a three-dimensional nanonetwork morphology. ACS Appl. Mater. Interfaces.

[16] Lai C, Sun Y, Lin B (2019). Synthesis of sandwich-like porous nanostructure of supercapacitors. Mater. Today Energy.

[17] Gopi CVM, Vinodh R, Sambasivam S, Obaidat IM, Kalla RMN, Kim HJ (2019). One-pot synthesis of copper oxide–cobalt oxide core–shell nano cactus-like heterostructures as binder-free electrode materials for high-rate hybrid supercapacitors. Mater. Today Energy.

[18] Yu G, Hu L, Liu N, Wang H, Vosgueritchian M, Yang Y, Cui Y, Bao Z (2011). Enhancing the supercapacitor performance of graphene/MnO_2_ nanostructured electrodes by conductive wrapping. Nano Lett..

[19] Yuan C, Li J, Hou L, Zhang X, Shen L, Lou XW (2012). Ultrathin mesoporous NiCo_2_O_4_ nanosheets supported on Ni foam as advanced electrodes for supercapacitors. Adv. Funct. Mater..

[20] Zhang J, Chen Z, Wang Y, Li H (2016). Morphology-controllable synthesis of 3D CoNiO_2_ nano-networks as a high-performance positive electrode material for supercapacitors. Energy.

[21] Wei Q, Jiang Y, Qian X, Zhang L, Li Q, Tan S, Zhao K, Yang W, An Q (2018). Sodium ion capacitor using pseudocapacitive layered ferric vanadate nanosheets cathode. iScience.

[22] Liu C, Ren QQ, Zhang SW, Yin BS, Que LF, Zhao L, Sui XL, Yu FD, Li XF, Gu DM, Wang ZB (2019). High energy and power lithium-ion capacitors based on Mn_3_O_4_/3D-graphene as anode and activated polyaniline-derived carbon nanorods as cathode. Chem. Eng. J..

[23] Zhang J, Wang Y, Liao HJ, Yang TY, Chen Z, Yan X, Zhou Z, Lv H, Liu WW, Chueh YL (2020). Hierarchical Mn-doped Fe_2_O_3_@rGO hollow core-shell spheres for high-performance hybrid capacitor. Mater. Today Energy.

[24] Chen L, Li D, Zheng X, Chen L, Zhang Y, Liang Z, Feng J, Si P, Lou J, Ci L (2019). Integrated nanocomposite of LiMn2O4/graphene/carbon nanotubes with pseudocapacitive properties as superior cathode for aqueous hybrid capacitors. J. Electroanal. Chem..

[25] Iftikhar, H., Debananda, M, Ganesh, D., Charmaine, L., Saad, G.M., Mostafa, S.S., Jae, J.S. Different controlled nanostructures of Mn-doped ZnS for high-performance supercapacitor applications. *J. Energy Storage*, **32**, 101767 (2020).

[26] Teeparthi SR, Awin EW, Kumar R (2018). Dominating role of crystal structure over defect chemistry in black and white zirconia on visible light photocatalytic activity. Sci. Rep.

[27] Hui, L., Shuang, L., Zhiling, Z., Xiaonan, Ali, A., Nguyen, D. C. T., Cho, K. Y., Oh, W. C. A simple ultrasonic-synthetic route of Cu_2_Se-graphene-TiO_2_ ternary composites for carbon dioxide conversion processes*, Fullerenes Nanotubes Carbon Nanostruct. 26*(12), 827–836 (2018).

[28] Navı´o, J.A., Hidalgo, M.C., Colo´n, G., Botta, S.G., Litter, M.I. Preparation and physicochemical properties of ZrO_2_ and Fe/ZrO_2_ prepared by a sol-gel technique, *Langmuir 17,* 202–210 (2001).

[29] Wang Y, Lai W, Wang N, Jiang Z, Wang X, Zou P, Lin Z, Fan HJ, Kang F, Wong CP, Yang C (2017). A reduced graphene oxide/mixed-valence manganese oxide composite electrode for tailorable and surface mountable supercapacitors with high capacitance and super-long life. Energy Environ. Sci..

[30] Qu D, Feng X, Wei X, Guo L, Cai H, Tang H, Xie Z (2017). Synthesis of MnO nano-particle@Flourine doped carbon and its application in hybrid supercapacitor. Appl. Surf. Sci..

[31] Xi Z, Qiang C, Anqi W, Jian X, Shishan W, Jian S (2016). Bamboo-like composites of V_2_O_5_/polyindole and activated carbon cloth as electrodes for all-solid-state flexible asymmetric supercapacitors. ACS Appl. Mater. Interfaces.

[32] Shengyang D, Laifa S, Hongsen L, Gang P, Hui D, Xiaogang Z (2016). Flexible sodium-ion pseudocapacitors based on 3D Na_2_Ti_3_O_7_ nanosheet arrays/carbon textiles anodes. Adv. Funct. Mater..

[33] Jing Z, Guiling W, Kui C, Ke Y, Kai Z, Jun Y, Dianxue C, Wang HE (2020). Growing NiS_2_ nanosheets on porous carbon microtubes for hybrid sodium-ion capacitors. J. Power Sources.

